# Template-Free Self-Assembly of Two-Dimensional Polymers into Nano/Microstructured Materials

**DOI:** 10.3390/molecules26113310

**Published:** 2021-05-31

**Authors:** Shengda Liu, Jiayun Xu, Xiumei Li, Tengfei Yan, Shuangjiang Yu, Hongcheng Sun, Junqiu Liu

**Affiliations:** College of Material Chemistry and Chemical Engineering, Hangzhou Normal University, Hangzhou 311121, China; liushengda0801@foxmail.com (S.L.); xujiayun@jlu.edu.cn (J.X.); lixiumei_chem@163.com (X.L.); tengfeiyan@163.com (T.Y.); yusj@hznu.edu.cn (S.Y.)

**Keywords:** template-free self-assembly, 2D polymer, polymer capsules, polymer films, polymer tubes and rings

## Abstract

In the past few decades, enormous efforts have been made to synthesize covalent polymer nano/microstructured materials with specific morphologies, due to the relationship between their structures and functions. Up to now, the formation of most of these structures often requires either templates or preorganization in order to construct a specific structure before, and then the subsequent removal of previous templates to form a desired structure, on account of the lack of “self-error-correcting” properties of reversible interactions in polymers. The above processes are time-consuming and tedious. A template-free, self-assembled strategy as a “bottom-up” route to fabricate well-defined nano/microstructures remains a challenge. Herein, we introduce the recent progress in template-free, self-assembled nano/microstructures formed by covalent two-dimensional (2D) polymers, such as polymer capsules, polymer films, polymer tubes and polymer rings.

## 1. Introduction

Self-assembly is a common occurrence in nature and in living systems, including the double helix structure of DNA, the aggregation and folding of proteins, and cells or living organisms. Over the past decades, the implementation of nano/microstructured objects or materials using bioinspired self-assembled strategies is one of the exciting advances in the field of chemistry [1,2,3,4,5]. A diverse range of nano/microstructures, such as films [6,7,8,9], spheres [10,11,12,13], tubes [14,15,16,17], and cages [18,19] have been successfully constructed, and the common crucial aspect to their success is the employment of non-covalent interactions between the building blocks, allowing for dynamic, reversible and “self-error-correcting” procedures, which eventually result in the production of the most thermodynamically stable structures. However, the dynamic, weak and reversible characteristics of the non-covalent interactions in support structures also have the tendency to make structures less robust for practical applications [20]. Although several template-assisted covalent nano/microstructures have been explored recently, their preparation requires preorganization and removal of the template, which is time-consuming and sophisticated.

**Figure 1:**
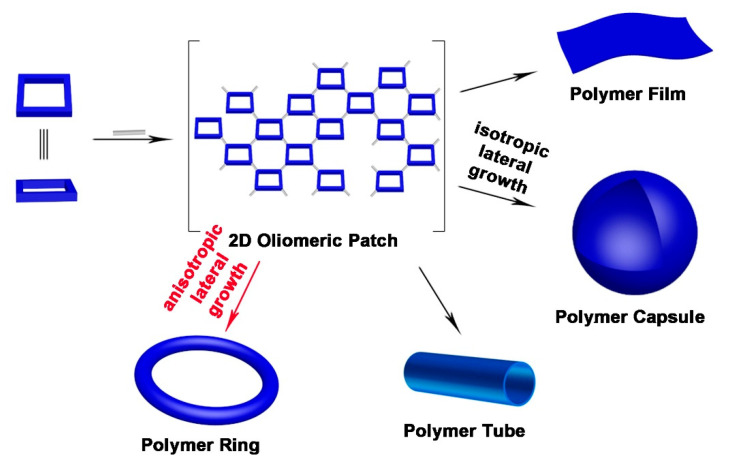
Template-free self-assembly of nano/microstructured materials by covalent 2D polymers.

Recent studies have shown that the polymerization of rigid, flat-shaped building blocks with multiple symmetrical polymerizable groups and linear linkers with double polymerizable groups can lead to a variety of well-defined nano/microstructures, rather than irregular polymers without template-assisted preorganization. In addition, the shape, size, properties and function of these nano/microstructured materials can be controlled through the rigorous and precise selection of parameters that include building blocks, linear linkers and reaction media. These building blocks first grow in polymerization into 2D oligomeric patches without the addition of preorganization or templates. Subsequently, further growth of 2D oligomeric patches to drive the system with or without curvature production forms polymer capsules, polymer films, polymer tubes and polymer rings. This review demonstrates the recent discoveries and advances in template-free self-assembly of structurally defined nano/micromaterials formed by 2D polymers (Figure 1).

## 2. Polymer Capsules

Polymer capsules, which are hollow, spherical shells made of polymers, have received extensive attention for their versatile applications in cargo encapsulation, drug delivery, imaging and biocatalysis [21,22,23,24]. Self-assembled strategies that rely on the balance of free energy and entropy of the system to obtain the thermodynamically most stable state of the polymer capsules are quite challenging.

**Figure 2:**
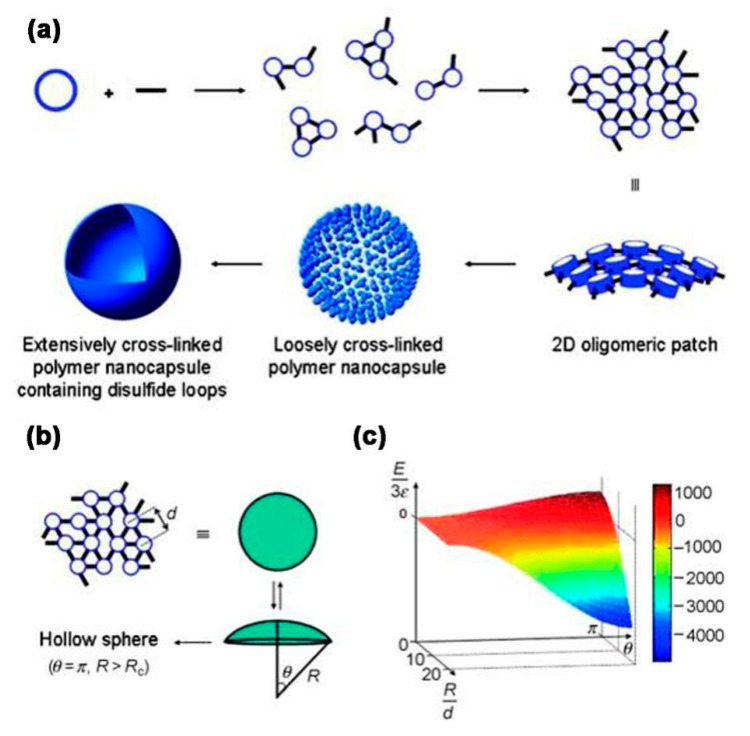
(**a**) The proposed mechanism of the formation of the polymer capsules; (**b**,**c**) theoretical model and energy profile of the capsule formation process. Reprinted with permission [25] Copyright 2007 Wiley-VCH.

**Figure 3:**
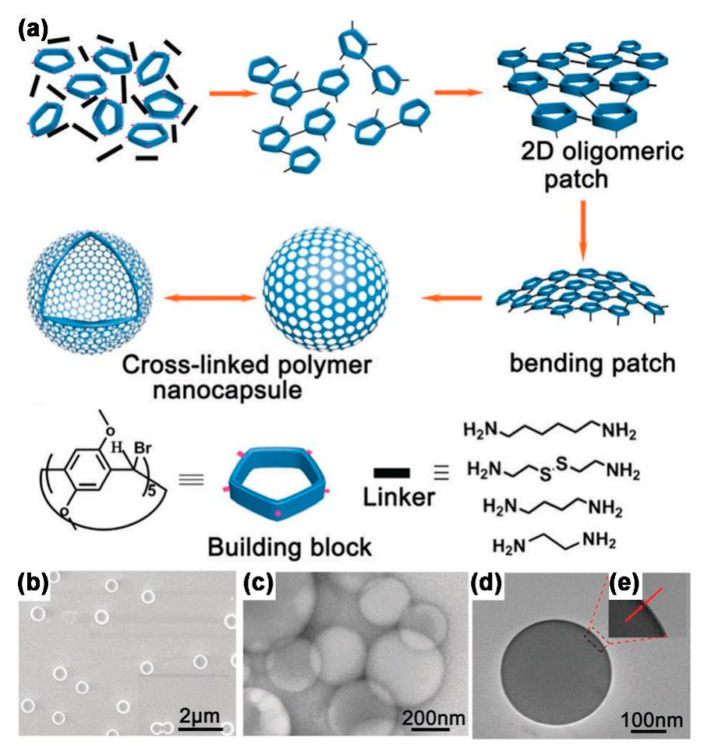
(**a**) Schematic representation of the preparation of the polymer capsules; (**b**) SEM; (**c**) TFM; (**d**,**e**) high resolution TEM images of polymer capsules. Reprinted with permission [27] Copyright 2017 Royal Society of Chemistry.

The first discovery for the preparation of polymer nanocapsules based on template-free self-assembled strategy that used supramolecular macrocyclic host molecules as building blocks was reported by Kim and co-workers in 2007 [25]. A monolayer thick polymer nanocapsule of a stable structure, narrow size distribution and regular morphology with a diameter of 110 nm and a wall thickness of 2.1 nm was successfully constructed by ultraviolet (UV) chemical polymerization by using cucurbit [6]uril (CB [6]) derivative (allyloxyCB [6]) as the building block and dithiol (3,6-dioxa-1,8-octanedi-thiol) as the linker. The mechanism of formation of nanocapsules is as follows (Figure 2): (1) The building blocks and the linkers were initially polymerized laterally to form a 2D oligomeric lamellar structure. (2) As the reaction continued, the oligomers continued to grow by lateral cross-linking, forming larger lamellar structures. When the lamellar structure reached a certain level of dimensional growth, it ceased to grow in a planar direction, and began to curl in order to reduce the stresses due to both solvent effects and thermodynamic effects. The curled lamellar structures could continue to react, resulting in a thermodynamically stable spherical product such as a loose capsule. (3) The unreacted reactive groups in the shell of the loose capsule were further cross-linked with the linker, which ultimately formed a well-matured, highly cross-linked and robust polymer nanocapsule. Theoretical calculations showed that the formation of capsules was mainly determined by the self-energy of the polymers in the reaction process, since the principle of minimum energy drove the curling of the lamellar structure into the capsule structure. In order to adequately test this mechanistic hypothesis, it was discovered that, by changing the solvent for assembly, the length of the linker, the capsule size could be controlled [26]. In addition to cucurbit [n]uril (CB [n]), pillar [n] arene (P [n] A) is a new type of macrocyclic host molecule with excellent host–guest chemistry properties. Liu and co-workers reported the first facile synthesis of laterally modified pillar [5] arene (P [5] A) derivative (bromoP [5] A) by methylene-bridged bromination reaction of P [5] A, which was used as a novel building block for the preparation of template-free, self-assembled polymer nanocapsules by the polymerization with diamines (Figure 3) [27,28,29]. Scanning electron microscopy (SEM) results showed that after polymerization, a large amount of spherical structures of uniform size and structure were produced, with the sizes between 300 and 500 nm. Transmission electron microscopy (TEM) results showed these spherical structures with a hollow structure inside, indicating the formation of highly ordered hollow capsules. High resolution TEM results also showed that the wall thickness of these hollow capsules was particularly thin, with a thickness of 1.2 nm. The results of the control experiments showed that the length of the cross-linker has a great influence on the formation of capsules, for which only an alkyl diamine of the suitable length should be selected. The longer the length of the cross-linker, the larger the size of the capsule formed. The polymer capsules were dispersible, uniform in shape, homogeneous and dimensionally controllable with an interior that can be well coated with cargos. The capsule surface can also be modified with functional groups by covalent and non-covalent approaches, providing the basis for the development of highly efficient smart materials.

**Figure 4:**
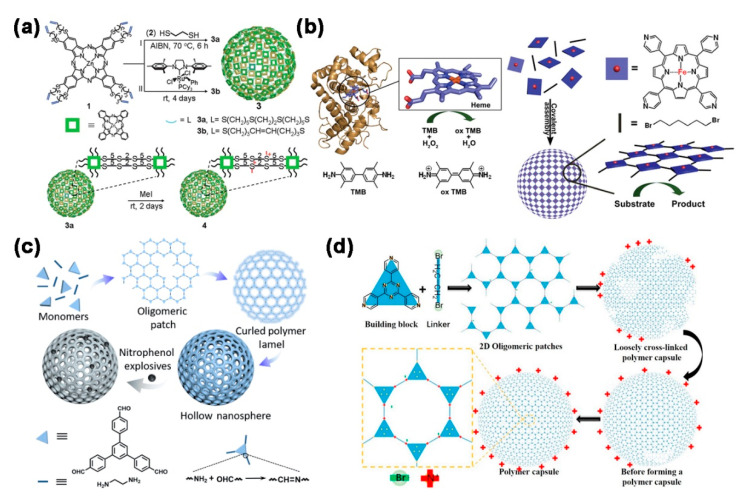
Schematic representation. (**a**) Direct synthesis of Pc polymer capsules. Reprinted with permission [38] Copyright 2013 Royal Society of Chemistry; (**b**) The active site of HRP and the synthesis of polymer capsules with peroxidase-like activity. Reprinted with permission [42] Copyright 2018 Royal Society of Chemistry; (**c**) The formation of the polymer capsules with positive charge. Reprinted with permission [49] Copyright 2020 Elsevier; (**d**) The formation of the AIE polymer capsules and the application in the detection of nitrophenol explosives. Reprinted with permission [46] Copyright 2020 Science China Press and Springer Nature.

The structure of the membrane layer of the polymer capsules plays a crucial role in the properties of the polymer capsules, which will further influence the final applications of polymer capsules. Other than the macrocyclic molecules, various symmetrical and functional molecules have been developed for the construction of polymer capsules. For instance, phthalocyanine (Pc) is a common photosensitizer (PS) which is traditionally used in the biomedical field for photodynamic therapy (PDT) [30,31,32,33]. With the intense and selective absorption of light in the area of 600–800 nm by Pcs, a high depth of light penetration in normal tissues is permitted while minimizing risks and complications such as burns [34,35,36,37]. Kim and co-workers fabricated Pc nanocapsules by the thermal polymerization of the building blocks of zinc (Zn) Pc derivative (ZnPc with eight olefin groups) and the linkers of 1,2-ethanedithiol. Further post-synthetic modifications of ZnPc nanocapsules were allowed to improve their dispersion in aqueous solution without changing the morphology of the nanocapsules or the characteristics of the ZnPc building block (Figure 4a) [38]. In particular, the ZnPc nanocapsules exhibited a higher efficiency of singlet oxygen generation (SOG) and in vitro photo-toxicity than the Pc monomers, indicating the potential of ZnPc nanocapsules as a PS for PDT. It is well-known that iron (Fe) porphyrin derivative (heme) is the active center of peroxidases that can reduce toxic reactive oxygen species, thus providing good protection to the organism [39,40,41]. Inspired by the structure of peroxidases, Liu and co-workers employed a peroxidase-active Fe porphyrin derivative (5,10,15,20-tetrakis (4’-pyridyl-Fe(III)-porphyrin) as the building block for the polymerization with 1,6-dibromohexane as the linker in N,N-dimethylformamide (DMF), which resulted in hollow nanocapsules with peroxidase-like activity (Figure 4b) [42]. The enzymatic test results showed that the optimum pH for catalysis was 4.0, similar to that of the natural horseradish peroxidase (HRP) and other peroxidase models. In addition, the enzyme model had high catalytic activity and a good affinity for the substrate. The first-order rate constant (k_cat_) and the apparent Michaelis constant (K_M_) for the enzyme model were 35.62 min^−1^ and 0.106 mmol^−1^ min^−1^, respectively, with an apparent secondary reaction rate constant of k_cat_/K_M_ = 3.36 × 10^5^ L mol^−1^ min^−1^. The K_M_ of this model was much smaller than that of natural HRP, suggesting that the constructed peroxidase model had a better substrate affinity for 3,3,5,5-tetramethyl-benzidine (TMB) than natural HRP. Although its secondary reaction rate constant was smaller than that of HRP, it was better than that of some of the reported artificial peroxidase models. In addition, glucose could be oxidized to gluconic acid and hydrogen peroxide (H_2_O_2_) by glucose oxidase, and H_2_O_2_ could further oxidize TMB to a blue product by the nanocapsules with peroxidase properties, which enabled the sensitive detection of low concentrations of glucose. The high specificity of glucose oxidase for glucose made it possible to selectively detect glucose from a wide range of sugars. In addition, quaternary ammonium-based polymers are usually positively charged, so they can interact with bacterial cell membranes to inhibit their growth [43,44,45]. The advantages of these charge-interactive materials are their chemical stability, low irritation, long-lasting inhibition and almost no resistance to bacteria. Liu and co-workers produced a polymer nanocapsule with positively charged pyridine quaternary ammonium by covalent polymerization using 2,4,6-tris(4-pyridyl)-1,3,5-triazine as the building block and 1,2-dibromoethane as the cross-linking linker (Figure 4d) [46]. Antibacterial experiment results showed that the positively charged polymer nanocapsules exhibited significant inhibitory effects on Gram-negative *Escherichia coli* DH5α (*E. coli*), with the minimum inhibitory concentration (MIC) and minimum bactericidal concentration (MBC) of 0.04 mg/mL and 0.1 mg/mL, respectively. SEM results showed that when *E. coli* and polymer nanocapsules were co-cultured for 1 day, the cell membrane of *E. coli* became rough and showed wrinkling, shrinkage or even fracture. The surface of *E. coli* as a control group was bright and smooth and showed no obvious signs of damage. Traditional antimicrobial materials such as comb or dendritic cationic polymers inhibited bacterial growth by adsorbing and inserting hydrophobic groups into the cell membrane, thereby disrupting the structure of the bacteria. The large size of the polymer nanocapsules, with a diameter of 200 nm, made it difficult to be effectively embedded in the phospholipid membrane. Therefore, it was thought that the nanocapsules were capable of inhibiting bacteria by physical adsorption of the positive charge on their surface and the negative charge on the surface of the bacterial cell membrane. What is more, fluorescent conjugated polymer nanomaterials with aggregation induced emission (AIE) properties offered the advantages of superior brightness, high uniformity of dispersion and outstanding optical stability for explosives detection [47,48]. To provide effective detection of nitrophenol explosives, Liu and co-workers prepared AIE-based fluorescent conjugated polymer nanocapsules with an amplified fluorescent signal response by utilizing the Schiff’s base reaction of the 1,3,5-tris(4-formyl-phenyl)benzene (TFPB) building block and the ethylenediamine linker (Figure 4c) [49]. According to the photoinduced electron transfer process, high emission nanocapsules in solution had a remarkably selective and sensitive quenching response to the nitrophenol explosives, achieving a Stern–Volmer constants (K_sv_) of 9.67 × 10^5^ M^−1^ for 2,4,6-trinitrophenol (TNP) and 3.14 × 10^5^ M^−1^ for 4-nitrophenol (NP), respectively. In addition, a simple test strip for the detection of TNP was produced with a detection limit as low as 10^−9^ M. This research provided an opportunity for the sensing and detection of fluorescent conjugated polymer nanomaterials with AIE properties.

## 3. Polymer Films

Polymer films are lamellar structures of flat monomers cross-linked by covalent bonds in a periodically ordered arrangement [50]. These polymer films usually exist in three forms: layered crystals, multilayer structures or monolayer structures. In an effort to better study the properties of 2D polymer films and to exploit their properties, obtaining single-molecule layer structures is attempted. In order to obtain single or few-layer structures, it is generally necessary to peel off the multilayer structures with the aid of solvents or external forces, or to introduce solid surfaces to assist in obtaining single-layer structures. However, the preparation of ultra-thin 2D polymer structures without the aid of any template or an interfacial force is still a great challenge.

**Figure 5:**
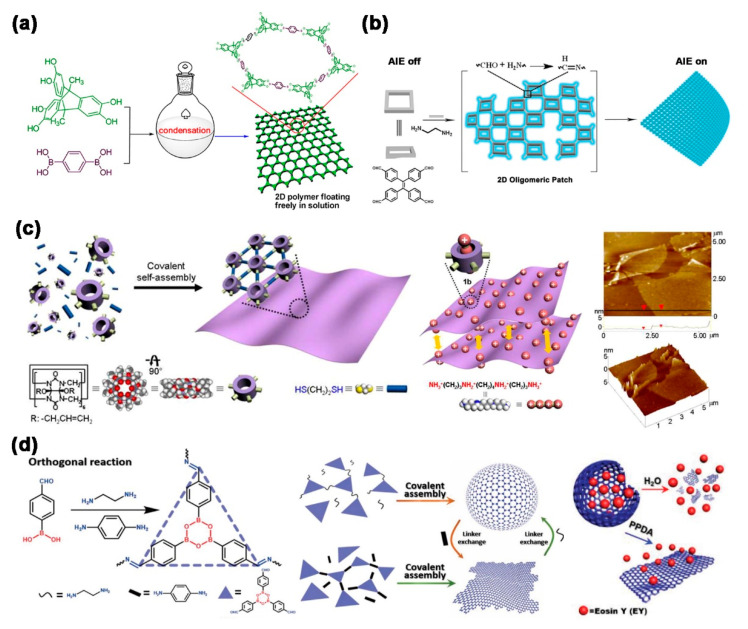
Schematic representation. (**a**) Direct synthesis of 2D polymer films. Reprinted with permission [51] Copyright 2013 American Chemical Society; (**b**) The proposed formation of 2D AIE polymer film. Reprinted with permission [53] Copyright 2019 Wiley-VCH; (**c**) The free-standing formation of 2D polymer films and AFM images of monolayer thick 2D polymer films. Reprinted with permission [52] Copyright 2013 American Chemical Society; (**d**) Morphological transformation between imine-boroxine hybrid polymer capsules and films and Eosin Y release under stimulation. Reprinted with permission [54] Copyright 2020 Wiley-VCH.

The self-assembled approach in which building blocks in solution can be directly linked by linkers into ultra-thin 2D polymer films without the use of any templates, offers the advantage of one-step synthesis, simple and convenient. A facile method for the direct preparation of monolayer 2D polymer films in solution without adding any templates was developed by Zhao and co-workers in 2013 (Figure 5a) [51]. The triptene derivative (triptycene tricatechol) was intelligently considered as the building block and 1,4-benzenediboronic acid was selected as the linker to fabricate ultra-thin 2D polymer films. The structure of the triptene derivative was unique in which the vertical arrangement of the benzene groups effectively reduced the π-π interaction between the layers and the introduction of the methyl groups further increased the distance between the layers, making it easy to obtain a single or few-layer 2D polymer film. Although the tendency of monolayers to aggregate was still not completely suppressed, this strategy indeed created the possibility of the existence of non-aggregating monolayers of 2D polymer films. In addition, Kim and co-workers fabricated an ultra-large 2D polymer film structure with lateral dimensions of tens of microns with the polymerization of the building block of CB [6] derivative (allyloxyCB [6]) and the linker of 1,2-ethanedithiol (Figure 5c) [52]. Normally, the lamellar structure layers were often stacked on top of each other. In order to obtain a 2D lamellar layer with a single molecular layer, a positive charge was introduced into the CB [6] building block through host-guest interactions. Due to the charge repulsion between the layers, 2D polymer films with a single molecular layer were obtained. Atomic force microscopy (AFM) results showed that the thickness of the constructed 2D film structure was only 2.0 nm, corresponding to the theoretical single-layer structure. In order to observe the structure of the 2D polymer films, small size gold nanoparticles were introduced to the 2D polymer films. TEM results showed that the gold nanoparticles were uniformly distributed on the 2D polymer, except for the defective positions, and each gold nanoparticle was surrounded by six other gold nanoparticles, in which the distance between them matched the distance between the regularly arranged CB [6]-based building blocks. This suggested that the 2D polymer films were formed by the ordered arrangement of the building block of CB [6]. What’s more, Liu and co-workers constructed ultra-thin high emission polymer films employing tetraphenylethylene (TPE) derivative (4,4′,4″,4‴-(ethene-1,1,2,2-tetrayl) tetrabenzaldehyde) as the building block with AIE property and ethylenediamine as the linker (Figure 5b) [53]. TEM and high resolution TEM results verified the ultra-thin sheet-like morphology of the polymer. The morphology of the films was also confirmed by AFM, which showed that the uniform height was approximately 0.9 nm, indicating that the films were probably monolayer. According to restricted intramolecular motion (RIM) property of TPE molecules, the cross-linked TPE polymer films showed a higher fluorescence intensity and fluorescence quantum yield than the TPE monomers. Due to the excellent optical properties, the high emission polymer films showed considerable potential applications in the field of organic photoelectrics. In the subsequent study, Liu and co-workers succeeded in constructing 2D polymer films with an orthogonal reaction of boric acid dehydration and Schiff’s base reaction (Figure 5d) [54]. In experiments, the precursor 4-formylphenylboronic acid was firstly used to construct the boroxine building blocks, which were then polymerized with the para-phenylenediamine linkers to form the 2D polymer film structure. AFM results showed that the films were relatively flat and uniform, with a thickness of only 0.8 nm, presumably as single molecular layer sheets. When an excess of ethylenediamine linkers was added to the solution of 2D film structures, the morphology of the assemblies gradually changed from films to capsules. This was due to the dynamic and reversible nature of the Schiff’s base reaction, where excess flexible ethylenediamine replaced rigid para-phenylenediamine and the flexible ethylenediamine linkers increased the curvature of the product structures, thus facilitating their transformation into hollow capsules. In addition, when an exceeding amount of para-phenylenediamine linkers were added again to the solution of capsules, the morphology of the assemblies gradually transformed from capsules to 2D film structures. The morphological transformation between capsules and films by linker exchanges was applied to encapsulate and release cargos by stimulus response. The dynamic commutability of boroxine and imine linkages is potentially useful for the applications of multi-stimulus responsive smart 2D polymer materials in diverse areas such as sensing, separation and detection.

## 4. Polymer Tubes and Rings

The synthesis of higher-order nano/microscale structures with well-defined sizes and stable shapes has been a major goal in both polymer chemistry and material science. In addition to the composition, the topology of these structures also plays an essential role in defining some properties of materials. The development of higher-order structures can significantly increase the comprehension of the relationships between structures and properties for real applications. Therefore, the generation of these complex structures is necessary, especially template-free assembled structures. However, the “bottom-up” fabrication of higher-order structures formed by covalent polymers with different morphology remains a challenge.

**Figure 6:**
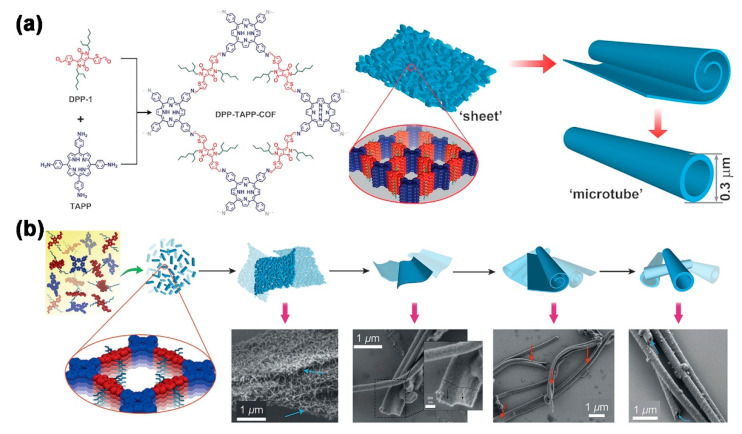
(**a**) Schematic representation of the synthesis of polymer tubes; (**b**) The proposed mechanism for tubular formation and SEM images of polymer structures at different time intervals. Reprinted with permission [55] Copyright 2018 Wiley-VCH.

In 2018, Beuerle and co-workers reported the first evidence for self-assembly of “bottom-up” microtubes based on covalent organic frameworks (COFs) that were obtained by the synthesis of the tetraphenylporphyrin (TPP) building blocks and the diketopyrrolopyrrole (DPP) linkers (Figure 6a,b) [55]. The available COF spontaneously aggregated into hollow microtubular assemblies with an outer diameter of approximately 300 nm and an inner diameter of around 90 nm. The mechanistic study showed a temporal dependence of the transition from initial 2D lamellar structures to tubular microstructures. It was proposed that automatic rolling into a tubular structure minimized unstable interactions of the solvent molecules. These discoveries pave the way for inventive future experiments with attractive single microtubules, including appropriate guest molecules as possible or even larger nano/microscale structures.

**Figure 7:**
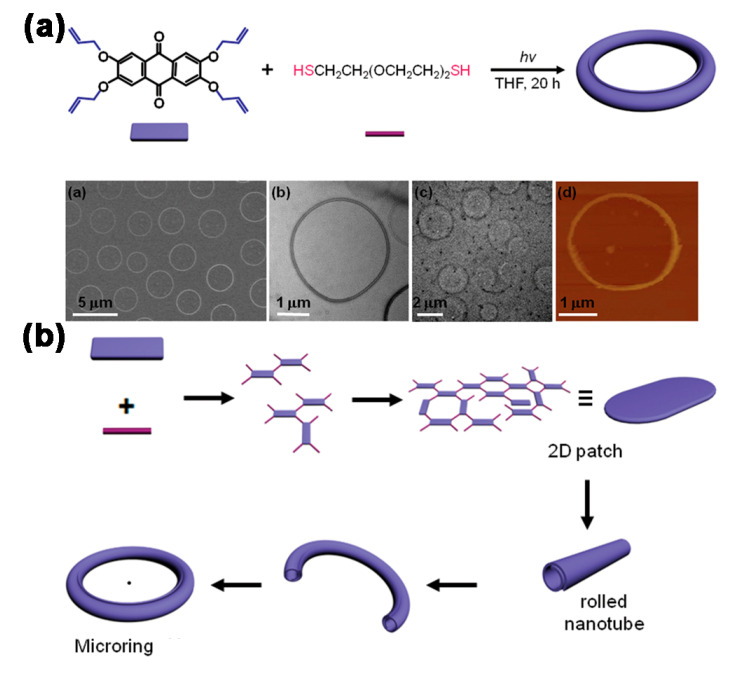
(**a**) Schematic representation of the synthesis of polymer rings and the SEM, AFM images; (**b**) The proposed mechanism for the formation of polymer rings. Reprinted with permission [57] Copyright 2015 American Chemical Society.

In addition, Kim and co-workers prepared hollow polymer rings with rectangular structures of tetrakis(allyloxy)anthraquinone in an anisotropic orientation as the building block and dithiol as the linker (Figure 7a,b) [56,57]. SEM, AFM and TEM results demonstrated the availability of hollow ring structures of uniform size and good dispersion with a diameter of 2.7 μm. It was shown that, with increasing concentration of the building blocks, the outer diameter of the ring structure became narrower, while the diameter of the cross section gradually increased. Further TEM results showed that the polymerization reaction began shortly after, with the appearance of elliptical 2D lamellar structure, and, as the reaction proceeded, the resulting lamellar structure gradually curled into a tubular structure, after which the tubular structure gradually bended and finally formed a hollow ring structure. It was presented that the generation of polymer rings was attributed to the anisotropic growth of rectangle-shaped building blocks. What is more, the polymer rings were used to encapsulate guest molecules, such as C_60_, or to provide a template for the fabrication of other structures. The novel approach exhibited promising potential in constructing hollow ring configurations and other associated structures of controlled sizes and chemical compositions.

## 5. Conclusions and Perspective

The self-assembly of molecules is one of the most important tools and technical methods for the creation of new substances. Thermodynamically controlled self-assembly can form the most energetically stable states. However, the lack of a detailed insight into the kinetics of the self-assembled procedure always hinders the design of kinetics-controlled self-assembled systems. In this review, we have introduced an unusual self-assembled strategy that breaks with traditional concepts of well-defined nano/microstructures formed by irreversible covalent 2D polymers. A variety of nanostructures, such as polymer capsules, polymer films, polymer tubes and polymer rings can be created by template-free self-assembly by means of proper design of building blocks, selective linkers and suitable use of reaction media. Rigid, flat-shaped building blocks with several symmetrical polymerizable groups and linear linkers are necessary for the formation of nano/microstructures. Multiple symmetrical polymerizable groups of rigid building blocks induce polymerization in the lateral dimension of the monomers. It enables the prevention of the normally expected random aggregate formation in a typical process for irreversible cross-linking of monomers, and allows the fabrication of an intermediate 2D oligomeric patch; a vital stage in the successful kinetic control of the self-assembled process. Significantly, the geometry of the building blocks and the reaction mediums have a major role that determines the ultimate size and shape of the nanostructures. The isotropic orientation of the building blocks leads to isotropic lateral growth of the intermediate 2D oligomeric patches, which ultimately results in the formation of polymer films in good solvents, while it leads to the production of polymer capsules and polymer tubes in poor solvents. In addition, the anisotropic orientation of the building blocks gives rise to anisotropic lateral growth of the intermediate 2D oligomeric patches, which ultimately generates the polymer rings.

This self-assembled strategy that uses covalent 2D polymers to form nano/microstructures offers new ideas for the construction of robust nano/microstructured materials. With the use of more multifunctional building blocks, such nano/microstructures can be extended to a wider range of applications. Furthermore, by endowing such nano/microstructures with stimulus-responsive properties, they were able to achieve intelligent and solid structured systems, when combined with the robust and responsive nature of the nano/microstructured materials. We envisage that the next studies will reveal the enormous untapped potential of the template-free self-assembled nano/microstructures formed by covalent polymers.

## Data Availability

Not applicable.
